# Retinal detachment in Type IX collagen recessive Stickler syndrome

**DOI:** 10.1038/s41433-024-03393-7

**Published:** 2024-10-15

**Authors:** Daniel Maghsoudi, Thomas RW Nixon, Howard Martin, Allan J Richards, Annie M McNinch, Philip Alexander, Arabella V Poulson, Martin P Snead

**Affiliations:** 1 Vitreoretinal Research Group, John van Geest Centre for Brain Repair, University of Cambridge, Forvie Site, Robinson Way, Cambridge, CB2 0PY UK; 2 NHS England Highly Specialised Stickler Syndrome Service, Cambridge University NHS Foundation Trust, Addenbrooke’s Hospital, Hills Road, Cambridge, CB2 0QQ UK

**Keywords:** Hereditary eye disease, Retinal diseases

## Abstract

**Objective:**

Stickler Syndrome (SS) is associated with eye, joint and orofacial abnormalities. Most cases are dominantly inherited through *COL2A1/COL11A1* variants encoding type-II/XI collagen, with patients having up to 78% retinal detachment (RD) risk. Rarer cases of recessive SS have also been identified, associated with pathogenic variants of genes including *COL9A1, COL9A2 & COL9A3* encoding type-IX collagen, but there is limited published data on patients’ phenotype or RD risk. Our study aimed to investigate RD risk in type-IX recessive SS, determining whether patients would benefit from prophylactic retinopexy. A secondary objective was to explore patient phenotypes, identifying key features which clinicians should identify, leading to earlier diagnosis.

**Methods:**

We report 13 cases from 11 families with Type-IX recessive SS, identified from the cohort attending the NHS England Highly Specialised Stickler Syndrome Service (1/1/15-31/12/22). Patients underwent multidisciplinary assessment by ophthalmology, rheumatology and audiology.

**Results:**

6/11 families exhibited previously undescribed genetic variants, and 7 had consanguineous parents. Clinical findings included abnormal vitreous architecture and high myopia. 15.4% of patients developed RD secondary to horseshoe retinal tears, with no cases of bilateral RD or giant retinal tears (GRTs). No patients had cleft palate, and 30.8% had midfacial hypoplasia. Hearing loss was more prevalent (91.7%) than in dominant SS. Arthropathy was uncommon but variable in manifestation.

**Conclusions:**

Ours results do not point to high RD nor GRT incidence in recessive SS, although given the rarity, our numbers are small. Prophylactic retinopexy should only be offered case-by-case for fellow eyes of patients presenting with GRT detachments in their first eye.

## Introduction

Stickler Syndrome (SS) is an inherited disorder associated with eye, ear, joint and orofacial abnormalities. Key ocular findings include congenital megalophthalmos, hypoplastic vitreous, paravascular lattice degeneration, quadrantic lamellar cataract and retinal detachment [1]. Other characteristic features include musculo-skeletal problems (arthralgia, hypermobility and early onset degenerative arthritis), hearing loss (both conductive and sensorineural), cleft palate and midfacial underdevelopment [2]. It is the most common cause of rhegmatogenous retinal detachment (RD) in children [3], and the most common heritable cause of retinal detachment. However, SS shows a highly variable phenotype both between and within families with the condition, and its inheritance can be both dominant or recessive through a variety of genes influencing the coding or assembly of type II, IX and XI collagen [1] as well as BMP4 (ref. [4]).

Correlation between genetic analyses and patient phenotypes shows that most SS exhibits autosomal dominant inheritance [5], with the most prevalent sub-group Type 1 SS associated with heterozygous *COL2A1* loss-of-function variants leading to deformities in Type II collagen [6]. These patients exhibit a pathognomonic membranous vitreous anomaly, congenital megalophthalmos and a very high (up to 78%) risk of retinal detachment [1]. Palate abnormalities are seen in 45% of patients [7] and hearing loss in 52% [8].

Type 2 SS also exhibits dominant inheritance and is caused by heterozygous *COL11A1* variants encoding the alpha-1 chain of Type XI collagen [9]. These patients have a different vitreous phenotype [10, 11] from Type 1 SS patients, with an apparently lower incidence of retinal detachment [12], a lower incidence of palate abnormalities [7] and greater likelihood of hearing loss [8]. Both subtypes are known to cause arthropathy with a wide-ranging phenotype [13, 14] and some experience severe arthropathy leading to functional impairment and requiring joint replacement [15, 16].

Whilst these two subtypes make up the majority of cases of Stickler Syndrome, several studies have identified cases of recessive SS, associated with both homozygous and compound heterozygous inheritance most commonly those encoding Type IX collagen, namely *COL9A1*, (ref. [17]) *COL9A2* (ref. [18]) and *COL9A3* (ref. [19]). To date, 22 patients from 12 families have been reported in the literature with variants in one of these three genes causing recessive SS [20]. These patients all had myopia (mostly >−6D high myopia), all had sensorineural hearing loss and most exhibited an abnormal, typically hypoplastic vitreous architecture. Retinal detachments were seen in 13.6% of patients, joint pain reported in 22.7%, and facial features in 40.9%. Cleft palate has not so far been reported in this sub-group. This phenotype is broadly similar to the features seen in autosomal dominant SS, but with significantly varying incidence of certain characteristics which may be important in directing diagnosis and patient management.

In this article, we report 13 patients from 11 families with recessive SS associated with type IX collagen variants. Our key objective is to investigate the incidence of retinal detachment to date in this novel, rare form of recessive SS to determine whether these patients would benefit from the prophylaxis protocol shown to be so effective in preventing GRT in type 1 SS [1].

## Materials and Methods

Patients were identified from the cohort attending the NHS England Highly Specialised National Stickler Syndrome Service based at Addenbrooke’s Hospital, Cambridge, UK between January 1^st^ 2015 – December 31^st^ 2022. Patients were included in the study if they had genetically confirmed autosomal recessive Stickler Syndrome as a result of pathogenic variants in COL9A1, COL9A2 or COL9A3. 13 patients from 11 families were identified and all underwent multidisciplinary assessment by ophthalmology, rheumatology and audiology. Patients underwent full ophthalmic assessment including refraction, slit-lamp biomicroscopy and vitreous and fundus examination alongside examination for systemic abnormalities of the face, palate, hearing and joints. X-ray findings were recorded in patients where radiology was indicated, and hearing was assessed by audiometry and tympanometry. Verbal informed consent was obtained from all patients where necessary and all data has been anonymised.

Molecular genetic analysis was performed by the East Genomics Laboratory hub, Addenbrookes’s Hospital, Cambridge UK. This comprised targeted Next Generation Sequencing on genes listed in the R45 Stickler syndrome panel (PanelApp, Genomics England [21]), enriched using a custom Twist Bioscience capture (Twist BioScience, San Francisco, USA) sequenced on an Illumina NovaSeq (Illumina, San Diego, California, USA). Variants were evaluated in accordance with guidelines from the American College of Medical Genetics and Genomics, the Association for Molecular Pathology [22] and the Association for Clinical Genomic Science guidelines version 4.01 [23].

## Results

Table 1 summarises the key clinical findings of all 13 patients. The patients had an age range of 3–42, with a median age of 14. All patients exhibited myopia, typically high myopia (>−6D), and 92.3% of patients exhibited an abnormal hypoplastic vitreous architecture. Retinal findings were wide-ranging, as described in Table 1. In 54% (*n* = 7) of patients no retinal abnormality was detected. Of the remaining patients, one had bilateral paravascular lattice degeneration (patient age 14), one showed snail track degeneration, three patients had peripheral retinal atrophy, and another had a dysplastic optic disc. Patient 7-I had undergone vitrectomy surgery for removal of an epiretinal membrane as well as cataract surgery in the right eye.

**Table 1:** Key Clinical Findings.

Number	Age	Gene	Variant	Consanguinity? (Y=Yes; N=No)	Refraction: RE/LE (D)	Retina	Vitreous	Face	Palate	Hearing	Joints	XR
1-I	12	COL9A1	c. 1519 C > T, p.(Arg507Ter)	Y (first cousins)	−8.5/−7	Normal	Hypoplastic	Normal	Normal	SNHL	No symptoms	Normal
2-I	20	COL9A1	c.1970delC, p.(Pro657fs) Concurrent ARID1B variant	N	−2/−4	RE: Peripapillary Atrophy, Dysplastic optic disk; LE: Peripapillary Atrophy	Normal	Normal	High arched	Severe mixed conductive/SNHL	No symptoms	Normal
3-I	7	COL9A2	c98delG, p.(Gly33AlafsTer53)	Y (first cousins)	−9/−9	Normal	Hypoplastic	Flat midface	Normal	HFSNHL	No symptoms	Normal
4-I	20	COL9A2	c. 1692_1717del p.(Gly565TrpfsTer32)	Y (first cousins)	−6.5/−7	Bilateral snail track degeneration, flat round holes	Hypoplastic	Normal	Normal	SNHL	Generalised pain - erythromelalgia	Normal
4-II	14	COL9A2	c. 1692_1717del p.(Gly565TrpfsTer32)	Y (first cousins)	−4/−2.25	Bilateral lattice degeneration	Hypoplastic	Normal	Normal	SNHL	Pain in legs	Refused
5-I	8	COL9A2	Compound heterozygous: c.1792+5 G > A; c.1918C>T p.(Arg640Ter)	N	−4.5/−5	Normal	Hypoplastic	Flat midface	Normal	SNHL	No symptoms	Normal
6-I	7	COL9A2	c.[75+2dup]; [75+2dup]	Y (first cousins)	−12.5/−12.75	Normal	Hypoplastic	Normal	High arched	SNHL	Features of Juvenile Idiopathic Arthritis (JIA) & Perthes	Features consistent with JIA, spondyloepiphyseal dysplasia
7-I	42	COL9A3	c.107_116del10, p.(Pro36fs) Concurrent COL2A1 AD Stickler	N	−15/−13	RE: ERM peel age 31; LE temporal chorioretinal peripheral atrophic areas temporally, intraretinal cystic change	Hypoplastic	Normal	Normal	Asymptomatic; No audiometry	No symptoms	Not commented
8-I	28	COL9A3	c. 1411 C > T, p. Arg471Ter	Y (first cousins)	−23/−23	Highly myopic appearance	Hypoplastic	Normal	Normal	HFSNHL	Severe arthropathy in shoulders and hip, requires wheelchair	Bones osteopenic, spinal scoliosis surgery, joint space narrowing in both knees
8-II	25	COL9A3	c. 1411 C > T, p. Arg471Ter	Y (first cousins)	−7/−7	LE: previous retinal detachment age 24 [horseshoe tear, subsequent recurrent subtotal RD]; RE: normal	Hypoplastic	Normal	Normal	HFSNHL	No symptoms	Mild scoliosis, otherwise normal
9-I	42	COL9A3	c. 1477 G > A, p.(Gly493Arg)	N	−4/−6	LE: previous retinal detachment age 36 (macula-off rhegmatogenous RD, no complications), atrophic patch superior to retina; RE: normal	Hypoplastic	Normal	Normal	Normal on audiometry	No symptoms	Features of possible collangenopathy
10-I	7	COL9A3	Compound heterozygous: c.1740delT (p.Gly581Aspfs*92); c.1933G>A (p.Gly645Arg)	N	−6.5/−6	Normal	Hypoplastic	Flat midface	Normal	SNHL	No symptoms	Normal
11-I	3	COL9A3	c. 1548 G > A p.(Pro516Pro)	N	−10/−10	Highly myopic appearance	Hypoplastic	Flat nasal bridge	Normal	SNHL	No symptoms	Normal
SUMMARY				Consanguinity: 7/13; 53.8%	High myopia (>−6): 10/13; 77%	Retinal Detachment: 2/13; 15.4%	Hypoplastic vitreous: 12/13; 92.3%	Midfacial hypoplasia: 4/13; 30.8%		Confirmed hearing problems in 11/12; 91.7%	Skeletal issues: 4/13; 30.8%	

2 of 13 patients (15.4%) had experienced retinal detachment requiring surgical repair (8-II; 9-I). These were both due to horseshoe tears, secondary to posterior vitreous detachment, occurring at the ages of 24 and 36 respectively. There was no involvement of the contralateral retina for either patient, and neither experienced a Giant Retinal Tear (GRT).

Facial abnormalities were present in 30.8%, all consisting of midfacial hypoplasia in keeping with the phenotype seen in all SS subtypes. No patients exhibited a cleft palate, but 15.4% of patients did have a high arched palate as is common to many other connective tissue disorders.

Most patients exhibited hearing loss, though the phenotype varied between patients. 7/13 had sensorineural hearing loss, 3/13 had high-frequency sensorineural hearing loss and one had severe combined conductive and sensorineural hearing loss requiring a bone-anchored hearing aid. Interestingly, one patient (9-I), had completely normal hearing confirmed on audiometry, and another (7-I) has no symptoms of hearing deficit but has not undergone audiometry testing.

Joint problems were experienced in 30.8% of patients. However, the phenotype of these varied significantly between individuals. Patient 4-II described pain in their legs after exercise which did not have a significant impact on their life, whilst patient 4-I described generalised pain with moderate impact on day to day living. More significant issues were seen in patient 6-I with diagnosed Juvenile Idiopathic Arthritis and features of spondyloepiphyseal dysplasia on imaging, and patient 8-I whose severe shoulder and hip arthropathy has left them requiring a wheelchair to mobilise, alongside findings of osteopenia and joint space narrowing in both knees on plain radiography.

### Genetic analysis

Genetic analysis for 6 of the patients identified variants in *COL9A1-3* genes which have not previously been reported. Genetic analysis for patient 1-I identified a c. 1519 C > T, p.(Arg507Ter) homozygous variant of *COL9A1* which was also described by Nikopoulos et al. (ref. [24]) and Nixon et al. (ref. [20]). A further three families were also included in Nixon et al. 2022 (3-I; 4-I/II; 8-I/II). Genetic analysis for patient 7-I identified a c.107_116del10, p.(Pro36fs) homozygous variant of *COL9A3* which was also recently described in an Iranian family with recessive SS by Rad et al. (ref. [25]). The other 6 of 11 families exhibited variants which we could not find described elsewhere in existing literature. Pedigree mapping found consanguinity in the majority of patients (7 of 13).

## Discussion

We report 13 patients from 11 families with Type IX collagen recessive SS, with our key finding being that 15.4% of patients developed retinal detachment, with no GRTs. This lack of GRTs, taken in isolation, would suggest that prophylactic retinopexy by 360 cryotherapy against giant retinal tear detachment is unlikely to have been necessary in these patients. However, more than half the patients in this series are under 18 years of age, with median age 14 and age range 3–242, so the lifetime risk of RDs and GRTs remains to be determined.

Notably, more than half of patients in this study had a normal retinal examination, and only one radial paravascular lattice degeneration. However, retinal abnormalities are often not detected until adulthood even in Type 1 dominant SS, and long-term follow of our patients is underway to determine whether they develop retinal abnormalities later in life.

Other clinical features including high myopia and a hypoplastic vitreous were seen in most of our patients, in keeping with findings from Nixon et al. (ref. [20]). Interestingly, the 30.8% incidence of facial abnormalities and lack of cleft palates in both our cohort and the wider literature may indicate a significant difference from autosomal dominant SS, where cleft palate is a relatively common finding either in isolation or as part of the Pierre Robin Sequence [14].

Hearing loss has previously been reported as a consistent feature of type IX collagen recessive SS, present for every patient with the condition [20]. Our cohort exhibited an 91.7% incidence of hearing loss, most commonly presenting as sensorineural hearing loss requiring hearing aids. However, some patients had completely normal hearing, suggesting hearing loss cannot be considered a consistent feature of the condition. It does appear to be more common in recessive SS than type 1 SS, where only half of patients show hearing impairment [8].

Joint problems were reported in 30.8% of patients in this study, broadly in line with the 22.7% incidence reported by Nixon et al. (ref. [20]) for recessive Stickler. However, it is important to note that joint pain can vary significantly between patients – the majority of patients in this series had no or minimal symptoms, whilst two had severe arthropathy. Whilst 8-I exhibited severe arthropathy requiring a wheelchair, their relative with an identical variant of COL9A3 (8-II) had no joint problems other than mild scoliosis, highlighting the phenotypic heterogeneity of recessive Stickler Syndrome. As a result, joint problems are an inconsistent diagnostic tool for recessive SS and correlate poorly with other non-rheumatological symptoms of the condition. Furthermore, it is again important to note that since the majority of our patients are under 18 (median age 14), it is possible that the incidence of joint problems would increase over time within the cohort. Type IX collagen recessive SS does appear to be associated with a lower incidence of skeletal abnormalities, with nearly 50% of children with dominant SS exhibiting abnormalities on knee and spinal radiographs [13]. The principal differences between autosomal dominant and recessive Stickler Syndrome, discussed above, are summarised in Table 2.

**Table 2:** Comparison of principal clinical features in AD and AR Stickler Syndrome.

Feature	Incidence in AD Stickler Syndrome	This study: AR Stickler syndrome (pending continued cohort analysis)
Retinal Detachment	Up to 78% [1]	15.4%
Palate abnormalities	45% [7]	0%
Hearing loss	52% [8]	91.7%
Joint abnormalities	42.6–45.9% [13]	30.8%
Myopia	85–90% [5, 16]	100%

This series suggests that recessive Stickler Syndrome may have a lower risk of retinal detachment than the more common dominant subtypes of the condition, but this can only be confirmed or refuted on further longitudinal study. Until such data is available, prophylactic retinopexy cannot be recommended based on current evidence, and should only be offered on a case-by-case basis with respect to fellow eyes of patients presenting with giant retinal tear detachments in their first eye. Our results also suggest that recessive SS is associated with similar facial abnormalities, but may have a lower incidence of cleft palate and arthropathy, but a higher incidence of hearing loss than dominant SS. Recognition of the distinct features of recessive SS are important in prompting clinicians to request an appropriate molecular genetic analysis, leading to early diagnosis and allowing patients to receive appropriate counselling and multi-disciplinary management. Given the current explosive development and delivery of genetic testing services, genetic testing is increasingly likely to become routine, and in fact all the genes for type IX recessive Stickler syndrome are already included in the Genomics England PanelApp [21], along with the genes for autosomal dominant Stickler syndrome.

Patients must also be warned of the early warning symptoms of posterior vitreous detachment and retinal detachment, enabling early surgical intervention as required.

A diagnosis of SS should be considered by clinicians seeing young patients with congenital hearing loss and high myopia, particularly recessive SS if there is a history of consanguinity. Early diagnosis is particularly important to reduce the risk of dual sensory impairment from blindness due to retinal detachment in addition to deafness, and so that appropriate education and development support can be put in place.

## Summary

### What was known before

- Stickler Syndrome is typically an autosomal dominant disease, with a few reported cases of recessively inherited Stickler Syndrome.
- These recessive patients may have similar symptoms to their autosomal recessive counterparts, but there is very little literature on their phenotype or risk of retinal detachment.

### What this study adds

- Determines the retinal detachment risk in patients with Type IX collagen recessive Stickler Syndrome, a rarer and poorly understood subtype of the disease, and determines that prophylactic retinopexy cannot be recommended based on current evidence, and should only be offered on a case-by-case basis with respect to fellow eyes of patients presenting with giant retinal tear detachments in their first eye.
- Describes the phenotype of these patients in greater detail than was previously available in the literature, highlighting key differences to AD Stickler, and describing key features which should prompt clinicians to consider sending patients for genetic testing for the disease.

## Data Availability

The data that support the findings of the study are not available, beyond what is included in the tables and manuscript, due to reasons of patient confidentially.
